# Inhibition of Oxygen-Induced Ischemic Retinal Neovascularization with Adenoviral 15-Lipoxygenase-1 Gene Transfer via Up-Regulation of PPAR-γ and Down-Regulation of VEGFR-2 Expression

**DOI:** 10.1371/journal.pone.0085824

**Published:** 2014-01-21

**Authors:** Zhi Li, Tao He, Ke Du, Yi-Qiao Xing, Yuan-Min Run, Ying Yan, Yin Shen

**Affiliations:** 1 Eye Center, Renmin Hospital of Wuhan University, Eye Institute of Wuhan University, Wuhan, Hubei, China; 2 Department of Ophthalmology, Hospital Affiliated to Hubei University of Arts and Science, Xiangyang Central Hospital, Xiangyang, Hubei, China; 3 Department of Oncology, Hospital Affiliated to Hubei University of Arts and Science, Xiangyang Central Hospital, Xiangyang, Hubei, China; 4 Clinical Laboratory, Hospital Affiliated to Hubei University of Arts and Science, Xiangyang Central Hospital, Xiangyang, Hubei, China; 5 Department of Ophthalmology, Wuhan General Hospital of Guangzhou Military, Wuhan, Hubei, China; Cedars-Sinai Medical Center; UCLA School of Medicine, United States of America

## Abstract

15-lipoxygenase-1 (15-LOX-1) plays an important role in angiogenesis, but how it works still remains a controversial subject. The aims of our study are focused on determining whether or not 15-LOX-1 inhibiting oxygen-induced ischemic retinal neovascularization (RNV) and the underlying regulatory mechanism involving of 15-LOX-1, peroxisome proliferator-activated receptor γ (PPAR-γ) and vascular endothelial growth factor receptor 2 (VEGFR-2) in oxygen-induced retinopathy (OIR). Recombinant adenoviral vectors that expressing the 15-LOX-1 gene (Ad-15-LOX-1-GFP) or the green fluorescence protein gene (Ad-GFP) were intravitreous injected into the OIR mice at postnatal day 12 (P12), the mice were sacrificed 5 days later (P17). Retinal 15-LOX-1 expression was significantly increased at both mRNA and protein levels after 15-LOX-1 gene transfer. Immunofluorescence staining of retinal sections revealed 15-LOX-1 expression was primarily in the outer plexiform layer (OPL), inner nuclear layer (INL) and ganglion cell layer (GCL) retina. Meanwhile, RNV was significantly inhibited indicated by fluorescein retinal angiography and quantification of the pre-retinal neovascular cells. The expression levels of PPAR-γ were significantly up-regulated while VEGFR-2 were significantly down-regulated both in mRNA and protein levels. Our results suggested 15-LOX-1 gene transfer inhibited RNV in OIR mouse model via up-regulation of PPAR-γ and further down-regulation of VEGFR-2 expression. This could be a potentially important regulatory mechanism involving 15-LOX-1, PPAR-γ and VEGFR-2 during RNV in OIR. In conclusion, 15-LOX-1 may be a new therapeutic target for treating neovascularization diseases.

## Introduction

Retinal neovascularization (RNV) plays a key role in many ocular neovascularization diseases pathological processes, such as proliferative diabetic retinopathy (PDR), “wet” age-related macular degeneration (AMD) and retinopathy of prematurity (ROP). In normal ocular tissues, RNV is regulated by two counter-balancing systems of pro-angiogenic factors and anti-angiogenic factors [1]. However, under some pathological conditions, this balance would be disrupted by up-regulation of pro-angiogenic factors and/or down-regulation of anti-angiogenic factors [2]. As a result, endothelial cell proliferation, migration, and tube formation leading to angiogenic growth of new blood vessels.

Lipoxygenases (LOXs) are important enzymes in lipid metabolism that catalyze the stereoselective dioxygenation of polyunsaturated fatty acids to their corresponding hydroperoxy derivatives [3], [4]. In mammals, LOXs are categorized with respect to their positional specificity of arachidonic acid oxygenation into 5-, 8-, 12-, and 15-LOXs [3], [4], [5]. 15-LOXs can be subclassified into 15-LOX-1 and 15-LOX-2 according to the specificity of tissue distribution and enzymatic characteristics [6] 15-LOX-1 is mainly expressed in reticulocytes, eosinophils, immature red blood cells, macrophages, airway epithelial cells and skin [7], [8], [9]. 15-LOX-2 expression is detected in prostate, lung, skin, and cornea [10]. In terms of enzymatic characteristics, 15-LOX-1 preferentially metabolizes linoleic acid primarily to 13-(S)-HODE and also metabolizes arachidonic acid to 15-(S)-HETE [9], [11]. On the other hand, 15-LOX-2 metabolizes arachidonic acid to 15-(S)-HETE but metabolizes linoleic acid rarely [6]. Previously, it was observed that 15-LOX-1 was involved in many pathological conditions, such as cell differentiation, inflammation, atherogenesis and carcinogenesis [12]. Recently, 15-LOX-1 has been implicated for their role in angiogenesis, but the role remains controversial and the underlying mechanism remains unclear [13].

Peroxisome proliferator-activated receptor γ (PPAR-γ) is a ligand-inducible transcription factor that belongs to the nuclear hormone receptor superfamily [14]. PPAR-γ expression is mainly found in brown and white adipose tissues, heart, skeletal muscle, colon, small and large intestines, kidney, pancreas, spleen and retina [15], [16]. Several natural compounds serve as endogenous ligands for PPAR-γ, such as linolenic acid, arachidonic acid and eicosapentaenoic acid [17]. In recent years, 15-LOX-1 and its metabolites have been reported to serve as endogenous ligands for PPAR-γ [18], but the regulatory mechanism remains unclear. Moreover, there has been increasing appreciation of the fact that PPAR-γ might be involved in the mechanisms that regulate angiogenesis. However, whether PPAR-γ receptors act as pro-angiogenic factors or anti-angiogenic factors still remain a very controversial issue [19].

Vascular endothelial growth factor (VEGF) has been demonstrated to play an important role in ocular neovascularization [20], [21] and signaling through VEGF receptors is necessary for RNV[22]. VEGF binds to four receptors: VEGFR-1/Flt-1, VEGFR-2/KDR/Flk-1, Flt-3/Flk-2 and VEGFR-3/Flt-4 [23], but VEGFR-2 mediates most of the VEGF effects on blood vessels [24]. Recent reports suggest that PPAR-γ ligands can reduce the VEGFR-2 expression and inhibit angiogenesis in vascular endothelial cells [25].

We have previously used a mouse model of oxygen-induced retinopathy (OIR) to explore the expression of 15-LOX-1 and PPAR-γ during RNV. Our experiment provided evidence that expression both of 15-LOX-1 and PPAR-γ decreased during RNV in OIR. Meanwhile, 15-LOX-1 and PPAR-γ expression was negatively correlated with the progression of RNV. Our study suggests possible negative regulatory roles of 15-LOX-1 and PPAR-γ in RNV progression. The goals of this study were to determine whether 15-LOX-1 gene transfer inhibiting RNV and explore the underlying regulatory mechanism involving 15-LOX-1, PPAR-γ and VEGFR-2 in OIR.

In our current study we showed, for the first time, that 15-LOX-1 gene transfer significantly inhibited RNV via up-regulation of PPAR-γ and further down-regulation of VEGFR-2 expression in OIR. This is a novel and potentially important regulatory mechanism during RNV in OIR. 15-LOX-1 may be a new therapeutic target in the treatment of ocular neovascularization diseases.

## Materials and Methods

### Ethics statement

This study was carried out in strict accordance with the recommendations in the Guide for the Care and Use of Laboratory Animals of Wuhan University. The protocol was approved by the Committee on the Ethics of Animal Experiments of Wuhan University. All surgery was performed under sodium pentobarbital anesthesia, and all efforts were made to minimize suffering.

### Production of recombinant adenoviral vectors expressing 15-LOX-1

**Figure 1:**
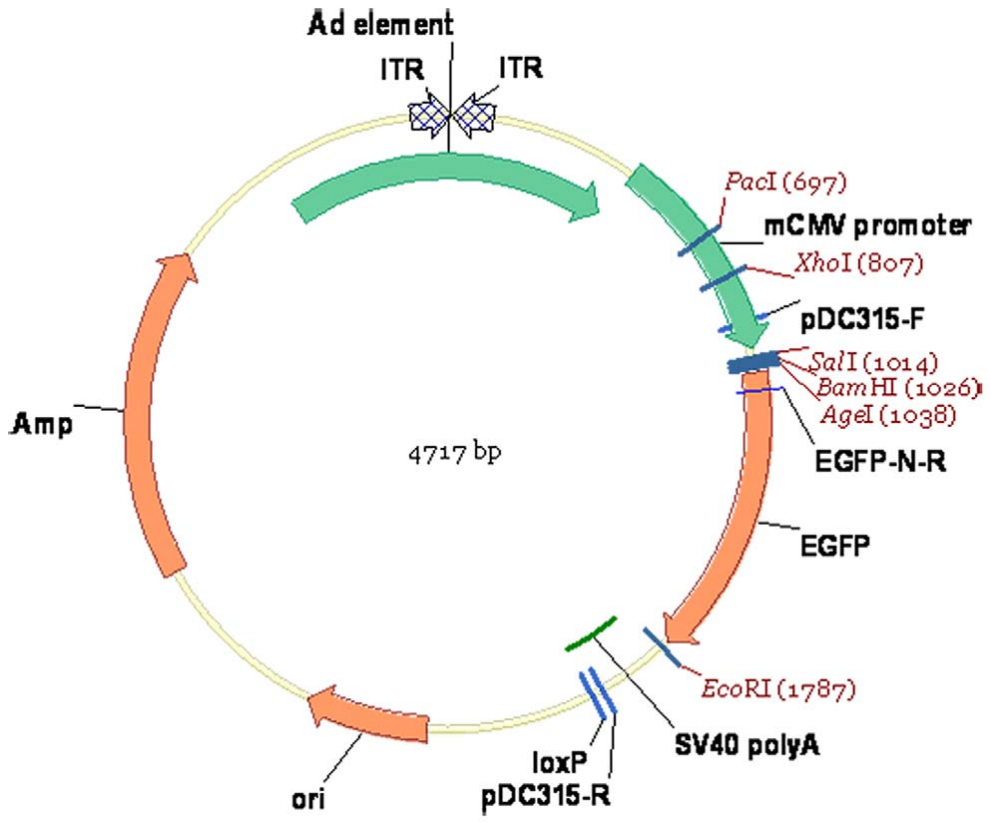
Schematic representation of the pDC315-EGFP plasmid is shown. ITR: inverted terminal repeat; Ad element: mouse 15-lipoxygenase-1 coding sequence; mCMV promoter: mouse cytomegalovirus immediate early promoter; EGFP: enhanced green fluorescent protein; SV40 poly A: SV40 polyadenylation signal; loxP: Eukaryotic restructuring enzyme (Cre) restructuring recognition sequence; Ori: E.coli origin of replication; Amp: ampicillin resistance sequence.

The recombinant adenoviral vectors that expressing the mouse 15-lipoxygenase-1 gene fused with GFP (Ad-15-LOX-1-GFP) was generated by AdMax**™** (Microbix Biosystems Inc. Canada). Briefly, the coding sequence of mouse 15-lipoxygenase-1 (m15-LOX-1) was amplified by RT-PCR. The primer sequences were as 15-LOX-1: (forward) 5′-GAG GAT CCC CGG GT ACC GGT CGC CAC C ATG GGT GTC TAC CGC ATC C-3′ and (reverse) 5′-TCA CCA TGG TGG CG ACC GGT TAT GGC CAC GCT GTT TTC C-3′. The PCR fragments and the pDC315-EGFP plasmid (Shanghai Genechem Company Ltd., China, Figure 1) were digested with the restriction endonuclease of Age I and then ligated with T4 DNA ligase to construct plasmid pDC315-15-LOX-1-EGFP. The pDC315-15-LOX-1-EGFP plasmid with a cDNA encoding m15-LOX-1 was used to co-transfect HEK293 cells with the helper plasmid pBHGloxΔE1, 3Cre (Microbix Biosystems Inc. Canada). Transfection solutions were prepared by mixing 5 µg of the pDC315-15-LOX-1-EGFP plasmid, 5 µg of the pBHGloxΔE1, 3Cre plasmid and 10 µl Lipofectamine 2000 in 50 µl antibiotics-free DMEM. After 8 d, the supernatant was harvested from HEK293 cells. After 2 rounds of virus amplification, the supernatant was filtered at 0.45 µm, and purified using Adeno-X™ virus purification kit (Clontech Laboratories, Mountain View, CA, USA). After resuspension, serially diluted adenovirus was used to transduce HEK293 cells. After 7 d, labeled HEK293 cells were counted to calculate the viral titer. The recombinant adenoviral vectors that expressing the green fluorescence protein gene (Ad-GFP) was used as a control. The viral titer of Ad-15-LOX-1-GFP and Ad-GFP were 1.25×10^11^ and 2.50×10^11^ plaque formation unit (PFU)/ml, respectively. Working solution was prepared to make 1.0 µl of the vehicle contained approximately 1.0×10^9^ PFU.

### Animal model of oxygen-induced retinopathy

Pregnant female C57BL/6J mice were provided by the Laboratory Animal Center of Wuhan University and were fed with a standard rodent diet (Test Diet®AIN-93G Growth Purified Diet) through pregnancy and lactation. Oxygen-induced retinopathy model was performed on C57BL/6J mice according to the method of Smith et al [26] with some modifications [27]. Briefly, at postnatal day 7 (P7), pups with their mothers were exposed to hyperoxia (75±2% O_2_) for 5 days (P7–P12) and then returned to normoxia (room air). Neovascularization occurs when returning to normoxia and peaks at postnatal day 17 (P17).

The mice were randomly divided into five experimental groups: (1) OIR mice treated with Ad-15-LOX-1-GFP group (OIR-LOX-GFP), (2) OIR mice without treatment group (OIR), (3) normal mice without treatment group (Control), (4) OIR mice treated with Ad-GFP group (OIR-GFP) and (5) normal mice treated with Ad-15-LOX-1-GFP group (LOX-GFP). OIR was induced in C57BL/6J mice from P7–P17. An intravitreous injection of 1.0 µl of the vehicle containing approximately 1.0×10^9^ PFU of Ad-15-LOX-1-GFP or Ad-GFP at P12 in Group 1, 4, 5 as indicated before. All mice were sacrificed at P17, one retina was collected from each mouse for biochemical assays while another eyeball was harvested as a whole for morphological study.

### Intravitreous injections of recombinant adenoviral vectors in mice

Intravitreous injections were administered with a Harvard pump microinjection apparatus and pulled glass micropipets, as previously described [28], [29]. Briefly, under a dissecting microscope, the sharpened tip of a micropipette was passed through the sclera, just behind the limbus into the vitreous cavity. Each micropipet was calibrated to deliver 1.0 µl of the vehicle containing approximately 1.0×10^9^ PFU of Ad-15-LOX-1-GFP or Ad-GFP.

### Immunofluorescence photograph after intravitreous injections of Ad-15-LOX-1-GFP

Mice were sacrificed 5 days after intravitreous injections of Ad-15-LOX-1-GFP or Ad-GFP at P12, and retinas were used to make cryosections for immunofluorescence staining. The procedures were performed following the protocol described before [30]. Posterior eyecups were fixed in 4% paraformaldehyde in 0.1 M phosphate buffer (PB, pH 7.4) for 20 minutes and chilled sequentially in 10% (w/v), 20% and 30% sucrose in 0.1 M PB at 4°C. The eyecups were then embedded in optimal cutting temperature (OCT, Miles Inc, Elkhart, IN, USA), frozen in liquid nitrogen, sectioned vertically at 14 µm thickness on a freezing microtome (Leica, Nussloch, Germany). The sections were mounted on gelatin-coated slides. Immunofluorescence staining was performed as described previously [31] with some modifications. In brief, deparaffinized and rehydrated cryosections were exposed to hydrogen peroxide to eliminate endogenous peroxide activity. The sections were then permeabilized for 30 minutes using PBS/0.4% Triton X-100 followed by blocking in PBS/5% BSA for 20 minutes. After 3×10 minutes washes with PBS, the sections were incubated with a mouse monoclonal antibody to 15-LOX-1 (Abcam, Cambridge, MA, USA) at 4°C overnight. The sections were then rinsed in PBS/0.2% Triton X-100 and incubated with a Cy3-conjugated goat anti-mouse IgG (1∶500, Jackson Immunoresearch Lab. Inc, West Grove, PA) for 1 hour. We omitted primary antibody (15-LOX-1) or secondary antibody for control experiments. The stained sections were rinsed again and mounted in a mounting medium containing 4′, 6-diamidino-2-phenylindole (DAPI). The slides were viewed at 400× magnification with a Nikon Eclipse Ti-SR fluorescence microscope (Nikon, Tokyo, Japan).

### Qualitative assessment of retinal neovascularization by fluorescein retinal angiography

Fluorescein retinal angiography was performed as previously described [26]. Briefly, at P17 mice were anesthetized and perfused via the left ventricle with 50 mg/ml high molecular weight (2×10^6^) Fluorescein isothiocyanate-dextran (FITC-dextran, Sigma). The mice were immediately sacrificed, and the eyes were enucleated and fixed in 4% paraformaldehyde for 3 hours. The retina was flat-mounted on a gelatin-coated slide. Quantification of RNV was performed as described previously [32], [33]. Briefly, images of the retina were taken at 40× magnification with an OLYMPUS BX41 fluorescence microscope (Olympus, Tokyo, Japan) and imported into Adobe Photoshop. Neovascular tuft formation was quantified by comparing the number of pixels in the affected areas with the total number of pixels in the retina. The avascular area in the retina was measured in the same way.

### Quantitative assessment of retinal neovascularization by counting pre-retinal neovascular cells

17-day-old mice were anesthetized and perfused transcardially with PBS. The eyes were fixed in Bouin's solutoin for 4 hours and stored in 70% ethanol for further experiments. After fixation, the eyes were embedded and cross-sectioned vertically through the center of the cornea and optic nerve. For quantification of pre-retinal neovascular cells, retinal structures were analyzed on 6 µm H&E-stained sections as described previously [26]. The pre-retinal neovascular cell nuclei were counted on eight discontinuous sections per eye and compared with the result between five different groups.

### Western blot analysis

The retina samples were homogenized in 200 µl of lysis buffer (20 mmol/l Tris, pH 7.4, 150 mmol/l NaCl, 1 mmol/l EDTA, 1 mmol/l orthovanadate, 1 mmol/l phenylmethylsulfonyl fluoride, 1 µg/ml leupeptin and 10 µg/ml aprotinin). After centrifugation at 12,000×g for 5 minutes at 4°C, the supernatant was collected. The protein concentration of the sample was determined using the Bradford assay. Equal amounts (40 µg) of proteins from each sample were loaded on SDS-PAGE and then transferred onto PVDF membranes at 200 mA for 1 hour. After blocking of nonspecific binding sites with 5% skim milk for 1 hour, the membrane was incubated with the primary antibodies as follows: mouse monoclonal antibody to 15-LOX-1 (1∶500 dilution, Abcam, Cambridge, MA, USA), mouse monoclonal antibody to PPAR-γ (1∶500 dilution, Santa Cruz Biotechnology, Santa Cruz, CA) and rabbit polyclonal antibody to VEGFR-2 (1∶1500 dilution, Abcam, Cambridge, MA, USA) overnight at 4°C. Antibody dilutions were made with a solution of 5% skim milk/0.1% Tris-buffered saline Tween-20. Then, membranes were incubated with horseradish peroxidase (HRP)-conjugated goat anti-mouse or rabbit IgG (1∶3000 dilution, Santa Cruz Biotechnology, Santa Cruz, CA) for 1 hour at room temperature. After three washes, the proteins were visualized with enhanced chemiluminescence (Amersham Biosciences, Piscataway, NJ, USA) detection. ß-actin staining served as the internal standard for all membranes. The experiments were repeated for five times.

### Real-time PCR

Total RNA was extracted from the frozen retina tissue by using Trizol reagent according to the manufacturer's instructions. cDNA synthesis was conducted according to the RNA PCR kit protocol (Takara, Dalian, China). ß-actin was used as a normalizing control. The primer sequences were 15-LOX-1: 5′-TTG GTT CTA CTG GGT TCC TAA TG-3′ (forward) and 5′-GGA GCC AAA CGA CAT TTA TCT G-3′ (reverse); PPAR-γ: 5′-ATG GAG CCT AAG TTT GAG TTT G-3′ (forward) and 5′-CAG CAG GTT GTC TTG GAT GTC-3′ (reverse); VEGFR-2: 5′-TCG AGC CCT CAT GTC TGA AC-3′ (forward) and 5′-TGA TGC TGT CCA AGC GTC TT-3′ (reverse); ß-actin: 5′-CTG AGA GGG AAA TCG TGC GT-3′ (forward) and 5′-CCA CAG GAT TCC ATA CCC AAG A (reverse). The PCR reaction was performed in a volume of 25 µl using SYBR green mix (Toyobo, Shanghai, China) on the Rotor-Gene 3000 Real-time PCR instrument (Corbett Research, Sydney, Australia). The thermal cycling program consisted of 1 minute at 95°C, 40 cycles of 15 seconds at 95°C, 15 seconds at 58°C, 45 seconds at 72°C. Relative quantification of the gene expression was performed using the 2^(−ΔΔCT)^ method [34]. All experiments were carried out three times.

### Statistical analysis

All values were given as means ± standard deviation (SD). Group differences were analyzed with one-way ANOVA followed by the Bonferroni post-test. P values less than 0.05 were considered statistically significant.

## Results

### Efficacy of 15-LOX-1 gene transfers into the mouse retina

**Figure 2:**
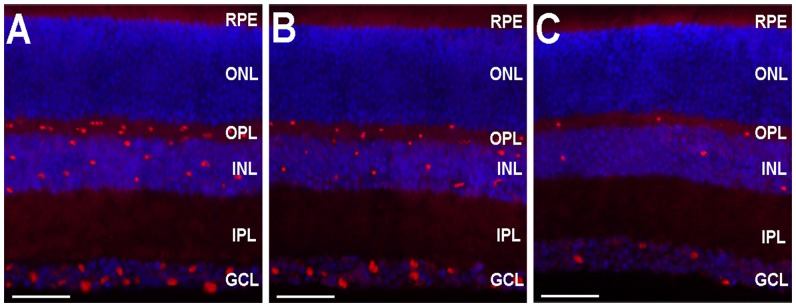
Expression of 15-LOX-1 gene transfers into the mouse retina. This figure shows the expression of 15-LOX-1 gene transfer in the retinas of 12-day-old C57BL/6J mice, 5 days after intravitreous injections of approximately 1.0×10^9^ plaque formation unit of Ad-15-LOX-1-GFP or Ad-GFP. Retinas obtained from LOX-GFP group, OIR-LOX-GFP group and OIR-GFP group mice were immunostained with an antibody for 15-LOX-1. Immunofluorescence staining indicated that the 15-LOX-1 protein is more strongly expressed in retinas from LOX-GFP group (A) and OIR-LOX-GFP group (B) mainly in the outer plexiform layer (OPL), inner nuclear layer (INL) and ganglion cell layer (GCL) than in retinas from OIR-GFP group (C). Cell nuclei were stained with 4′, 6-diamidino-2-phenylindole (DAPI). Images A-C were taken at 400×magnification for optimal comparison, scale bars = 35 um.

The efficacy of 15-LOX-1 gene transfer was assessed by immunofluorescence staining. Immunofluorescence staining of retinal sections revealed 15-LOX-1 expression was clearly shown, primarily in the outer plexiform layer (OPL), inner nuclear layer (INL) and ganglion cell layer (GCL) retinas of the intravitreous injections of Ad-15-LOX-1-GFP mice (Figure 2A and 2B), whereas mice intravitreous injections of Ad-GFP showed a small amount of fluorescence in the OPL, INL and GCL retinas (Figure 2C). In our control experiment of omitting the primary antibody (15-LOX-1) or omitting secondary antibody, no positive staining was presented (data not shown).

**Figure 3:**
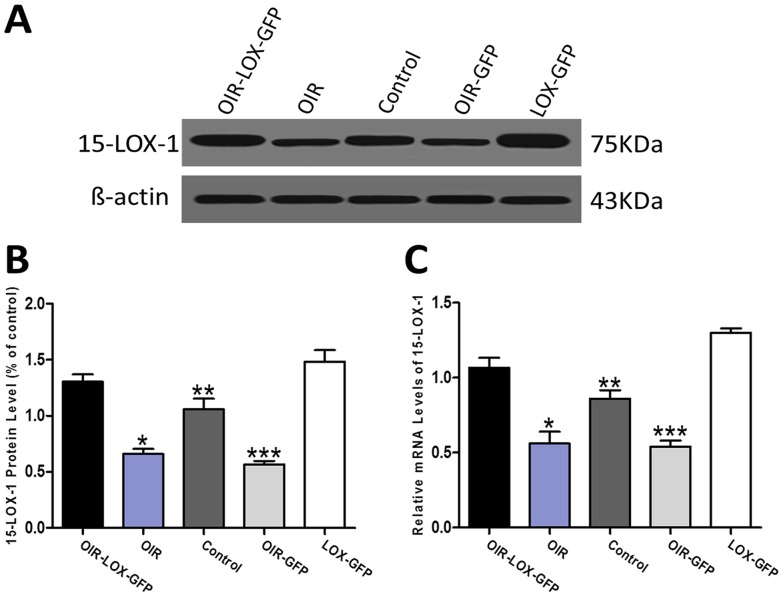
Efficacy of 15-LOX-1 gene transfers into the mouse retina. The efficacy of 15-LOX-1 gene transfers in the retinas of 12-day-old C57BL/6J mice, 5 days after intravitreous injection of approximately 1.0×10^9^ plaque formation unit of Ad-15-LOX-1-GFP or Ad-GFP. A, B: Western blot analysis confirmed that protein levels of 15-LOX-1 were higher in OIR-LOX-GFP group mouse retinas than those in OIR group, Control group and OIR-GFP group mouse retinas. The bands were quantified by densitometry and normalized by ß-actin, data were analysis as means±SD. OIR-LOX-GFP group versus OIR group *p<0.01; OIR-LOX-GFP group versus Control group **p<0.01; OIR-LOX-GFP group versus OIR-GFP group ***p<0.01, n = 5. C: Real-time PCR analysis further showed that mRNA levels of 15-LOX-1 in OIR-LOX-GFP group mouse retinas were higher than those in OIR group, Control group and OIR-GFP group mouse retinas. Data were shown as means ± SD, the relative amount of mRNA was normalized to ß-actin. OIR-LOX-GFP group versus OIR group *p<0.01; OIR-LOX-GFP group versus Control group **p<0.01; OIR-LOX-GFP group versus OIR-GFP group ***p<0.01, n = 3.

Western blot analysis showed that the 15-LOX-1 protein levels were substantially higher in LOX-GFP group (1.479±0.06) and OIR-LOX-GFP group (1.291±0.08) than those in Control group (1.071±0.07), OIR group (0.679±0.09) and OIR-GFP group (0.644±0.08) (p<0.01, n = 5) (Figure 3A and 3B).

Real-time PCR assays further confirmed that the mRNA expression levels of 15-LOX-1 in LOX-GFP group (1.300±0.03) and OIR-LOX-GFP group (1.067±0.07) were also higher than those in Control group (0.860±0.06), OIR group (0.560±0.08) and OIR-GFP group (0.540±0.04) (p<0.01, n = 3) (Figure 3C).

### Effects of 15-LOX-1 gene transfer on oxygen-induced ischemic retinal neovascularization

**Figure 4:**
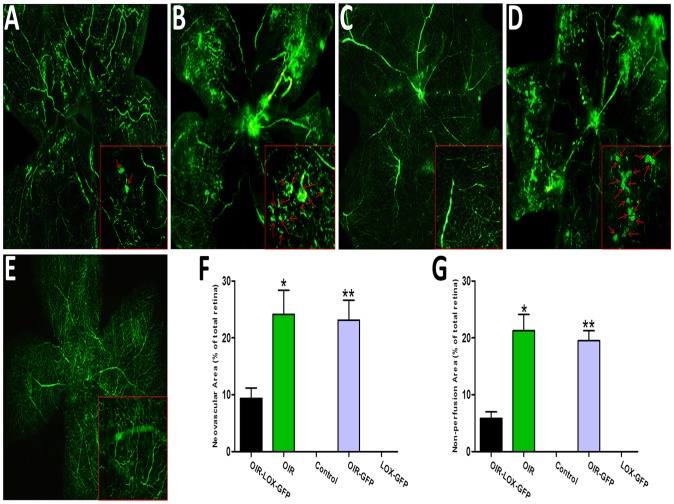
Effects of 15-LOX-1 gene transfer on retinal neovascularization in the oxygen-induced ischemic retinopathy model. Mice were perfused with fluorescein isothiocyanate-dextran at postnatal day 17 and the retina flat mounted. A: OIR-LOX-GFP group, B: OIR group, C: Control group, D: OIR-GFP group, E: LOX-GFP group. Magnification×40. The red rectangles in each figure are the magnification of randomly selected foci of the retina, and the red arrows indicate the neovascular tufts. F: Quantification of the neovascularization in the retinas from OIR-LOX-GFP group, OIR group, Control group, OIR-GFP group and LOX-GFP group mice. Retinal neovascularization was quantified by measuring the ratio of the neovascular tuft area to the total retinal area using the Image-Pro Plus 6.0 software (means ± SD). OIR-LOX-GFP group versus OIR group *p<0.05; OIR-LOX-GFP group versus OIR-GFP group **p<0.05, n = 8. G: Retinas non-perfusion areas were measured and compared with OIR-LOX-GFP group, OIR group, Control group, OIR-GFP group and LOX-GFP group using the Image-Pro Plus 6.0 software (means ± SD). OIR-LOX-GFP group versus OIR group *p<0.05; OIR-LOX-GFP group versus OIR-GFP group **p<0.05, n = 8.

The impact of 15-LOX-1 gene transfer on oxygen-induced ischemic RNV was evaluated in the OIR model, which develops oxygen-induced ischemic RNV [26]. To determine whether 15-LOX-1 gene transfer suppresses the oxygen-induced ischemic RNV, we examined the retinal vasculature in all five groups by using fluorescein angiography in retinal flat-mounts at P17. The results revealed significant neovascularization in the flat-mounted retinas from OIR group and OIR-GFP group mice (Figure 4B and 4D). In contrast, the retinas from OIR-LOX-GFP group mice developed less severe neovascular tufts (Figure 4A). Control group and LOX-GFP group mice maintained in room air showed no neovascularization in the retinal vasculature (Figure 4C and 4E). The RNV was quantified by measuring areas of neovascular tufts in retinal whole mounts, which showed that the retinas from OIR-LOX-GFP group mice have significantly smaller retinal neovascular tufts areas, approximately 39% of those in OIR group and 41% in OIR-GFP group respectively (Figure 4F). To further determine whether 15-LOX-1 gene transfer ameliorates the extent of hyperoxia-mediated vaso-obliteration in the OIR, we measured the non-perfusion areas in the central retina at P17. In OIR-LOX-GFP group, the non-perfusion areas were significantly smaller than those in OIR group (p<0.05, n = 8) and OIR-GFP group (p<0.05, n = 8) (Figure 4G). No obvious retinal inflammation, toxicity or other damage related to intravitreal injections were found in the dosage we used (Figure S1 and S2). Taken together, our results suggested that 15-LOX-1 gene transfer inhibited oxygen-induced ischemic RNV and ameliorated hyperoxia-mediated vaso-obliteration.

### Reduced pre-retinal neovascular cells in the oxygen-induced retinopathy model

**Figure 5:**
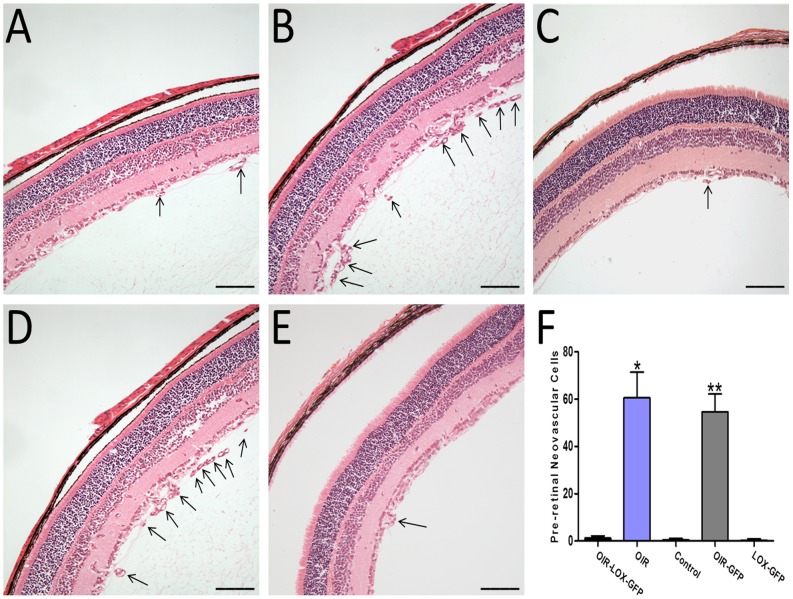
Effects of 15-LOX-1 gene transfer on pre-retinal neovascularization in mice with oxygen-induced ischemic retinopathy. At postnatal day 17, the eyeballs of OIR-LOX-GFP group, OIR group, Control group, OIR-GFP group and LOX-GFP group mice were fixed, sectioned, and stained with H&E. A: OIR-LOX-GFP group, B: OIR group, C: Control group, D: OIR-GFP group, E: LOX-GFP group. Arrows indicate pre-retinal neovascular cells. Magnification×200. Scale bar = 50 um. F: The average numbers of pre-retinal neovascular cells (means ± SD) of OIR-LOX-GFP group, OIR group, Control group, OIR-GFP group and LOX-GFP group mice using one-way ANOVA test. Pre-retinal neovascular cells were counted on eight non-continuous sections per eye and averaged. OIR-LOX-GFP group versus OIR group *p<0.05; OIR-LOX-GFP group versus OIR-GFP group **p<0.05, n = 8.

To further confirm the effect of 15-LOX-1 gene transfer on RNV, we quantified pre-retinal neovascular cells, a characteristic feature of the OIR model [26], [35]. The pre-retinal neovascular cells grown into the vitreous humor were counted on eight non-continuous cross-sections from each eye following an established method [26]. As shown in the Figure 5, the numbers of pre-retinal neovascular cells in the retinas from OIR-LOX-GFP group (1.25±0.89) were obviously lower than those in the retinas from OIR group (60.63±10.82) and OIR-GFP group (54.63±7.63) respective (P<0.05, n = 8), confirming the anti-neovascularization effect of 15-LOX-1 gene transfer in the retina.

### 15-LOX-1 gene transfer up-regulates the expression of PPAR-γ and down-regulates VEGFR-2 in the oxygen-induced retinopathy model

**Figure 6:**
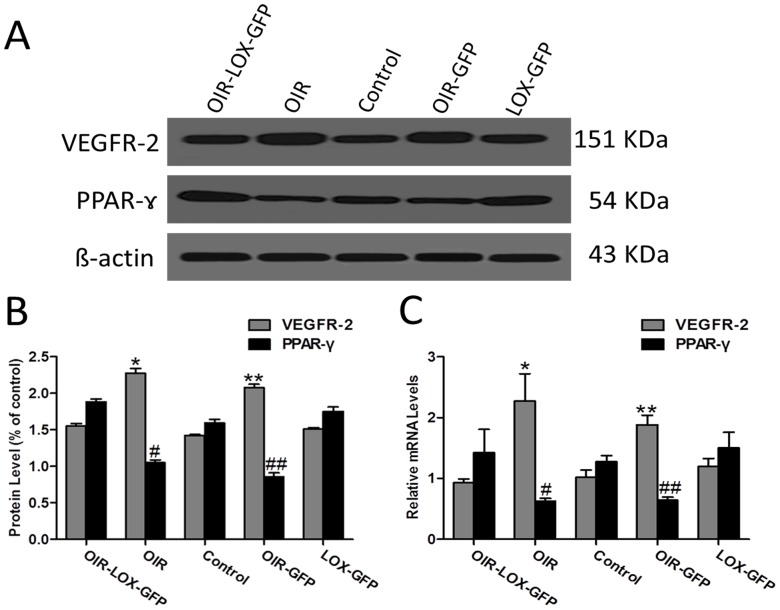
Effects of 15-LOX-1 gene transfer on retinal levels of PPAR-γ and VEGFR-2. OIR-LOX-GFP group, OIR group, Control group, OIR-GFP group and LOX-GFP group mice were euthanized, perfused, and eyeballs were enucleated at postnatal day 17. A, B: Retinal protein levels of PPAR-γ and VEGFR-2 were measured by Western blot analysis. Western blot analysis demonstrated that protein levels of PPAR-γ were down-regulated in OIR group and OIR-GFP group, and up-regulated in OIR-LOX-GFP group and Control group. On the contrary, protein levels of VEGFR-2 were up-regulated in OIR group and OIR-GFP group, but down-regulated in OIR-LOX-GFP group and Control group. OIR-LOX-GFP group versus OIR group *p<0.05; #p<0.05, OIR-LOX-GFP group versus OIR-GFP group **p<0.05; ##p<0.05, n = 5. C: Real-time PCR analysis of PPAR-γ and VEGFR-2 expression further showed that mRNA levels of PPAR-γ were significantly increased in OIR-LOX-GFP group and Control group, and decreased in OIR group and OIR-GFP group, whereas mRNA levels of VEGFR-2 were significantly increased in OIR group and OIR-GFP group, and decreased in OIR-LOX-GFP group and Control group. OIR-LOX-GFP group versus OIR group *p<0.05; #p<0.05, OIR-LOX-GFP group versus OIR-GFP group **p<0.05; ##p<0.05, n = 3.

To investigate the inhibitory mechanism of 15-LOX-1 gene transfer during RNV in OIR, we compared the expression levels of PPAR-γ and VEGFR-2 in all groups. As shown by Western blot analysis, protein levels of PPAR-γ were down-regulated in OIR group and OIR-GFP group, whereas up-regulated in Control group and OIR-LOX-GFP group. On the contrary, protein levels of VEGFR-2 were up-regulated in OIR group and OIR-GFP group, but down-regulated in Control group and OIR-LOX-GFP group (Figure 6A and 6B). Moreover, RT-PCR further demonstrated the above changes that mRNA levels of PPAR-γ were significantly increased in Control group and OIR-LOX-GFP group, and decreased in OIR group and OIR-GFP group. Whereas mRNA levels of VEGFR-2 were significantly increased in OIR group and OIR-GFP group, and decreased in Control group and OIR-LOX-GFP group (Figure 6C). Taken together, these results suggested that 15-LOX-1 gene transfer has an inhibitory effect on RNV in the OIR model via up-regulation of PPAR-γ and down-regulation of VEGFR-2 expression.

## Discussion

Retinal neovascularization (RNV) occurs in a number of ocular neovascularization diseases at all ages such as retinopathy of prematurity (ROP) in children, proliferative diabetic retinopathy (PDR) in young adults and age-related macular degeneration (AMD) in the elderly, which could lead to blindness if leave untreated [36], [37]. For many years, treatment for RNV of ocular neovascularization diseases was limited to laser photocoagulation and cryosurgery of the avascular zone, which effective for reducing severe vision loss, whereas could lead to serious side effects, including diminished night vision, reduced peripheral vision, and decreased visual acuity. It is urgent and important to develop efficacious therapies for the pathological angiogenesis. In recent years, anti-angiogenic strategies involving intravitreal injections have emerged as valuable new therapies for ocular neovascularization diseases [38], [39], [40], [41].

Accumulating evidence indicates that lipoxygenase pathways in general and 12/15-LOX in particular play a key role in the development of RNV during OIR [42]. There are two kinds of 15-LOX isozymes: 15-LOX-1 and 15-LOX-2. 15-LOX-1 has been implicated in angiogenesis, but previous studies apparently contradicted on the angiogenic properties of 15-LOX-1, varied from promoting angiogenic effect [43] to its suppression effect of angiogenesis [44], [45], [46]. The role and mechanism of 15-LOX-1 in oxygen-induced ischemic RNV have not been lucubrated previously. In the present study, we have been using fluorescein angiography on retinal whole mounts and quantified by counting pre-retinal neovascular cells to evaluate the effect of 15-LOX-1 gene transfer on RNV in the OIR mouse model. The results showed that the oxygen-induced ischemic RNV, as quantified by neovascular tufts, avascular areas and pre-retinal neovascular cells, were all suppressed in OIR-LOX-GFP group compared with those in OIR group and OIR-GFP group. Our studies have confirmed that 15-LOX-1 gene transfer in the retina inhibits oxygen-induced ischemic RNV. These observations indicate that 15-LOX-1 functions as an anti-angiogenic factor in the retina.

Peroxisome proliferator-activated receptor gamma (PPAR-γ) is a member of a ligand-activated nuclear receptor superfamily and plays a critical role in a variety of biological processes, including adipogenesis, glucose metabolism, and angiogenesis [47]. In recent years, 15-LOX-1 and its metabolites have been reported to serve as endogenous ligands for PPAR-γ, and 15-LOX-1 activates PPAR-γ through its metabolite 13-(S)-HODE [48], [49], [50]. Meanwhile, some studies have reported that PPAR-γ ligands are potent inhibitors of angiogenesis in vitro and in vivo [51], [52], [53], [25], [54], but other studies instead provided evidence that the same molecules may have important pro-angiogenic effects [55]. So far, the relationship between 15-LOX-1 and PPAR-γ during RNV in OIR has not been well-elucidated, and whether PPAR-γ receptors act as pro-angiogenic factors or anti-angiogenic factors still remain unclear. Recent studies demonstrated that PPAR-γ is expressed in normal retina, particularly in blood vessels and Müller cells, and that it is suppressed in experimental models of OIR [56]. At the same time, some studies demonstrated that the putative role of long-chain polyunsaturated fatty acids (LCPUFAs)-PPAR relationships in the retina [57] and direct PPAR-γ mediated effects of dietary ω-3-PU FAs on retinal vessel formation in an OIR model of pathologic retinal angiogenesis [58], [59]. Consistent with these studies, in our previous work, we found that both 15-LOX-1 and PPAR-γ expression were down-regulated in the development of RNV in a mouse model of OIR, following similar time-dependent variable tendency, which suggests possible negative regulator roles for 15-LOX-1 and PPAR-γ in RNV. In the present study, we found that the mRNA and protein expression levels of 15-LOX-1 and PPAR-γ were also up-regulated in OIR-LOX-GFP group. These results confirmed that 15-LOX-1 and PPAR-γ function as anti-angiogenic factor in the retina. The increased 15-LOX-1 levels may be due to the exogenous 15-LOX-1 gene transfer. The transcriptional and translational levels of PPAR-γ were significantly up-regulated by increasing the expression of 15-LOX-1. So we proposed the activated effect of 15-LOX-1 which serves as an endogenous ligand for PPAR-γ on retinal PPAR-γ expression. On the contrary, some previous reports have suggested that the down-regulation expression of PPAR-γ via increased expression of 15-LOX-1 [60], [61], [45], [46] and the mechanism involving the MAPK-dependent PPAR-γ phosphorylation was activated, which caused a down-regulation or loss of PPAR-γ transcriptional activity. Therefore, we propose there is a balance between these two opposing effects may determine the role of PPAR-γ receptor.

Angiogenesis is a multistep process that includes the degradation of basement membrane, proliferation, migration, and tube formation by endothelial cells; the process is stimulated by a variety of growth factors and cytokines [62], and among those, VEGF which signals mainly through the receptor tyrosine kinases VEGFR-1 and VEGFR-2, plays a dominant role in angiogenesis, with VEGFR-2 mediates most of the VEGF effects on blood vessels [24]. Previous studies revealed that PPAR-γ ligands reduce VEGFR-2 expression indirectly via suppression of the interaction between Sp1 and VEGFR-2 promoter region [63], consistent with the finding that there is no consensus PPRE sequence in the promoter region of the VEGFR-2 gene. Similarly, repression of Sp1-dependent binding and transactivation may be responsible for the reduced expression of VEGFR-2 upon PPAR-γ activation [64]. Agreed with the theory that the pro-angiogenic factor VEGFR-2 transcriptional activity could be inhibited by PPAR-γ activating, intravitreal 15-LOX-1 gene transfer efficiently inhibited the expression of VEGFR-2 in our study showing that 15-LOX-1 gene transfer can exert an anti-angiogenic effect by increasing PPAR-γ expression leading to down-regulating VEGFR-2 expression.

In conclusion, we have shown that 15-LOX-1 gene transfer inhibits RNV in a mouse model of OIR. The mechanism for the inhibition involves in activated effect of 15-LOX-1 which serves as an endogenous ligand for PPAR-γ on retinal PPAR-γ expression, and then further down-regulate the expression of VEGFR-2. Better understanding of the mechanism of function of 15-LOX-1 may provide a new therapeutic target in the treatment of ocular neovascularization diseases.

## Supporting Information

Figure S1 **Retinal inflammation after the intravitreous administration of Ad-15-LOX-1 at different doses.** 12-day-old C57BL/6J mice were treated with 1.0×10^8^, 1.0×10^9^ and 1.0×10^10^ PFU of Ad-15-LOX-1 via intravitreal injection. Five days after administration, all the animals were euthanized. The eyes were processed into paraffin, sectioned, and hematoxylin and eosin stained. Intravitreal injection of Ad-15-LOX-1 at dose of 1.0×10^8^ (A1–A2) or 1.0×10^9^ PFU (B1–B2) did not induce detectable inflammation (no detectable inflammatory infiltration cells, hemorrhagic inflammation and retinal edema) in the retina and vitreous cavity, but inflammatory reaction was observed at the dose of 1.0×10^10^ PFU (C1–C2). A1–C1 were taken at 200×, A2–C2 were taken at 400×.
(TIF)

Figure S2 **Retina TNF-α and ICAM-1 expression were detected after intravitreal injection of 1.0×10^8^, 1.0×10^9^ and 1.0×10^10^ PFU of Ad-15-LOX-1.** 12-day-old C57BL/6J mice were treated with 1.0×10^8^, 1.0×10^9^ and 1.0×10^10^ PFU of Ad-15-LOX-1 via intravitreal injection. Five days after administration, all the animals were euthanized. It is shown that the inflammatory response (indicated by TNF-α and ICAM-1 expression) induced by intravitreal injection of Ad-15-LOX-1 at dose of 1.0×10^8^ and 1.0×10^9^ PFU were obviously less than the dose of 1.0×10^10^ PFU.
(TIF)
