# Supplementary figures and images for: Inhibition of Oxygen-Induced Ischemic Retinal Neovascularization with Adenoviral 15-Lipoxygenase-1 Gene Transfer via Up-Regulation of PPAR-γ and Down-Regulation of VEGFR-2 Expression
Source: PLoS One. 2014 Jan 21;9(1):e85824. doi: 10.1371/journal.pone.0085824 (PMC3897531; doi:10.1371/journal.pone.0085824)

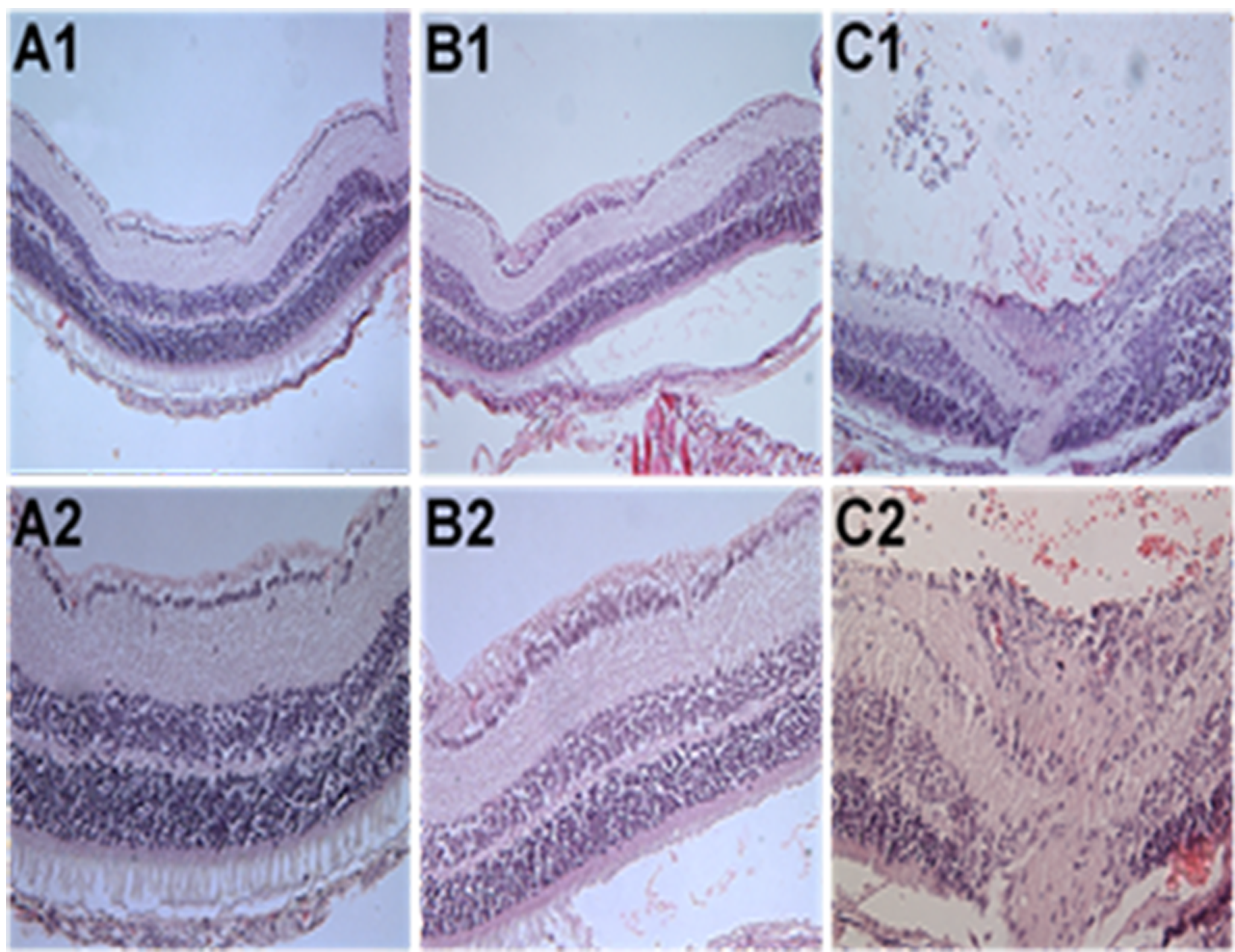

Supplement: Figure S1 — Retinal inflammation after the intravitreous administration of Ad-15-LOX-1 at different doses. 12-day-old C57BL/6J mice were treated with 1.0×10^8^, 1.0×10^9^ and 1.0×10^10^ PFU of Ad-15-LOX-1 via intravitreal injection. Five days after administration, all the animals were euthanized. The eyes were processed into paraffin, sectioned, and hematoxylin and eosin stained. Intravitreal injection of Ad-15-LOX-1 at dose of 1.0×10^8^ (A1–A2) or 1.0×10^9^ PFU (B1–B2) did not induce detectable inflammation (no detectable inflammatory infiltration cells, hemorrhagic inflammation and retinal edema) in the retina and vitreous cavity, but inflammatory reaction was observed at the dose of 1.0×10^10^ PFU (C1–C2). A1–C1 were taken at 200×, A2–C2 were taken at 400×. (TIF) [file pone.0085824.s001.tif]

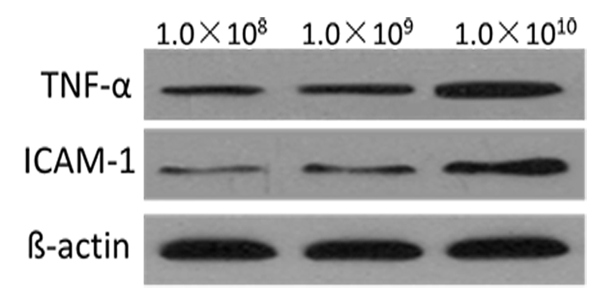

Supplement: Figure S2 — Retina TNF-α and ICAM-1 expression were detected after intravitreal injection of 1.0×10^8^, 1.0×10^9^ and 1.0×10^10^ PFU of Ad-15-LOX-1. 12-day-old C57BL/6J mice were treated with 1.0×10^8^, 1.0×10^9^ and 1.0×10^10^ PFU of Ad-15-LOX-1 via intravitreal injection. Five days after administration, all the animals were euthanized. It is shown that the inflammatory response (indicated by TNF-α and ICAM-1 expression) induced by intravitreal injection of Ad-15-LOX-1 at dose of 1.0×10^8^ and 1.0×10^9^ PFU were obviously less than the dose of 1.0×10^10^ PFU. (TIF) [file pone.0085824.s002.tif]
